# Survival results of a multicentre phase II study to evaluate D2 gastrectomy for gastric cancer

**DOI:** 10.1038/sj.bjc.6601761

**Published:** 2004-04-06

**Authors:** M Degiuli, M Sasako, A Ponti, F Calvo

**Affiliations:** 1 Department of Oncology, Division of Surgery, Via Cavour, 31, 10123 Turin, Italy; 2 National Cancer Center Hospital, Tokyo, Japan; 3 CPO Piemonte, ASL 1, Turin, Italy; 4 IGCSG include the following: Tiziano Allone, Dario Andreone, Alessandro Balcet, Riccardo Bussone, Marco Calgaro, Fabio Calvo, Lorenzo Capussotti, Maurizio Degiuli, Gianruggero Fronda, Marcello Garavoglia, Mauro Garino, Luigi Locatelli, Paolo Mello Teggia, Mario Morino, Fabrizio Olivieri, Fabrizio Rebecchi, Donatella Scaglione and Tito Soldati

**Keywords:** gastrectomy, lymph node dissection, survival, D2 gastrectomy

## Abstract

Curative resection is the treatment of choice for potentially curable gastric cancer. Two major Western studies in the 1990s failed to show a benefit from D2 dissection. They showed extremely high postoperative mortality after D2 dissection, and were criticised for the potential inadequacy of the pretrial training in the new technique of D2 dissection, prior to the phase III studies being initiated. The inclusion of pancreatectomy and splenectomy in D2 dissection was associated with increased morbidity and mortality. Following these results, we started a phase II trial to evaluate the safety and efficacy of pancreas-preserving D2 dissection. The results of this trial regarding the safety of pancreas preserving D2 dissection were published in 1998. In this paper, we present the survival results of this phase II trial to confirm the rationale of carrying out a phase III study comparing D1 *vs* D2 dissection for curable gastric cancer.

Italian patients with histologically proven gastric adenocarcinoma were registered in the Italian Gastric Cancer Study Group Multicenter trial. The study was carried out based on the General Rules of the Japanese Research Society for Gastric Cancer. A strict quality control system was achieved by a supervising surgeon of the reference centre who had stayed at the National Cancer Center Hospital, Tokyo, to learn the standard D2 gastrectomy and the postoperative management. The standard procedure entailed removal of the first and second tier lymph nodes. During total gastrectomy, the pancreas was preserved according to the Maruyama technique. Complete follow-up was available to death or 5 years in 100% of patients and the median follow-up time was 4.38 years.

Out of 297 consecutive patients registered, 191 patients were enrolled in the study between May 1994 and December 1996. The overall morbidity rate was 20.9%. The postoperative in-hospital mortality was 3.1%. The overall 5-year survival rate among all eligible patients was 55%. Survival was strictly related to stage, depth of wall invasion, lymph node involvement and type of gastrectomy (distal *vs* total).

Our results suggest a survival benefit for pancreas-preserving D2 dissection in Italian patients with gastric cancer if performed in experienced centres. A phase III trial among exclusively experienced centres is urgently needed.

Gastric cancer, which is the commonest cancer in Japan, remains a major cause of death also in Western countries. In Italy, it represents the third most frequent cause of death from cancer in both male and female patients (Decarli *et al*, 1998). Data from Italian Cancer Registries show a 27% 5-year survival rate (Rosso *et al*, 2001). This is consistent with other survival rates reported in Western countries. On the contrary, large retrospective Japanese series have shown significantly higher 5-year survival rates after radical gastrectomy. This impressive difference is largely related to earlier diagnosis, but it is possible that the more extensive lymph node dissection performed in Japan, where the stomach is usually removed along with the first and second tier nodal stations (D2 gastrectomy) (Sasako *et al*, 1997), also contributes.

Favourable patient survival after D2 gastrectomy has also been reported by some other non-Japanese retrospective nonrandomised trials (Pacelli *et al*, 1993; Siewert *et al*, 1993).

Nevertheless, the two large prospective randomised trials recently performed in the West (the MRC and the Dutch randomised surgical trials) failed to demonstrate a survival benefit for D2 gastrectomy as compared to D1 resection (Bonenkamp *et al*, 1999; Cuschieri *et al*, 1999). Furthermore, these trials showed a significant increase in post-operative morbidity and mortality after extended dissection.

These unfavourable results have been attributed mainly to the *en bloc* removal of the spleen and the tail of the pancreas for middle and upper third tumours in the D2 arms of both trials. Furthermore, the lack of experience in this technique of dissection and in postoperative care by each surgeon participating in these trials has been claimed as one of the reasons for the results (Bonenkamp *et al*, 1995; Cuschieri *et al*, 1996). Both studies were carried out without pretrial training and without preliminary studies to confirm the safety of the procedure locally, and were concluded before many surgeons would have reached the plateau of the learning curve.

The Italian Gastric Cancer Study Group (IGCSG) was set up in 1994 to confirm the safety and efficacy in survival of D2 resection with pancreas preservation, and a strict quality control system was implemented in a prospective one-arm phase II study. In 1998, we showed comparable postoperative morbidity and mortality rates with those reported after the standard resection, and documented that the D2 resection with preservation of the pancreas could be offered as a safe radical treatment of gastric cancer for Western patients in experienced centres (Degiuli *et al*, 1998).

We now report the survival data of the patients of the same trial.

## PATIENTS AND METHODS

### Eligibility and assessment of curability

Patients eligible for participation in this study were to have histologically proven and preoperatively potentially curable adenocarcinoma of the stomach. Patients who required emergency procedures, who harboured a coexisting cancer, who were >80 years old or who had a comorbid cardiorespiratory dysfunction that would preclude more extensive dissection were excluded. After preoperative staging to exclude clinical evidence of distant metastasis, all patients were registered and underwent staging laparotomy. Eligible cases were those without any evidence of peritoneal and/or liver metastasis, involvement of the oesophagus, cardias or duodenum, and biopsy-proven metastasis in para-aortic and/or retropancreatic nodes.

### Treatment

The surgical protocol was based on the general rules of the Japanese Research Society for Gastric Cancer (JRSGC, 1981a, 1981b). The D2 dissection entailed removal of the first and second tier nodes along with the lymph nodes of the left side of the hepatoduodenal ligament. During total gastrectomy, the spleen was removed while the tail of the pancreas was preserved according to the technique described by Maruyama *et al* (1995), unless it was suspected to be invaded by the tumour. In the case of a clinical T1 tumour, splenectomy was not carried out.

Distal gastrectomy was performed in cases of early gastric cancer (EGC) or well demarcated advanced gastric cancer (AGC), such as Borrman type 1 or 2, with a tumour-free margin of at least 2 cm, or in case of infiltrative AGC, type 3 or 4, with a tumour-free margin of at least 5 cm to the proximal resection line. A total gastrectomy was performed in all other cases.

For all enrolled patients, chemotherapy was not given until recurrence was diagnosed.

### Pathological classification

As compared with our previous papers, tumours were restaged according to the fifth edition of UICC TNM Classification of Malignant Tumours and the Japanese Classification of Gastric Carcinoma, 2nd English edition (UICC, 1997; JGCA, 1998).

### Quality control

A surgeon from the reference centre (MD) stayed at the National Cancer Center Hospital, Tokyo, to learn the D2 dissection from a specialist Japanese surgeon (MS). He was given didactic videos, papers and explanatory booklets edited by Japanese authors. MD became the supervisor of the trial.

The IGCSG was set up in April 1994 and nine institutions participated. Each centre had two surgeons attending all the operations.

Before starting the trial, several meetings were organised among participating centres to explain the terminology, to debate the proper indications and demonstrate the surgical technique. At least one of the two surgeons of each participating institution observed the first 10 procedures in this trial, which were performed at the reference centre. Afterwards, MD attended the first three operations performed at each institution.

### Registration

The study was organised and directed from a central office at the reference centre (Department of Oncology, Division of Surgery, Turin, Italy). Data on enrolment, surgical procedures, histopathologic findings, postoperative course and patient follow-up evaluation were collected by the surgeon at each institution and posted to the data centre at the central office. Patients were followed up at regular intervals: every 3 months during the first 2 years and every 6 months thereafter. In addition, an enquiry on vital status and cause of death was collected for all patients at the municipal roster office. The final follow-up date was 31 December 2002. Complete follow-up was available in 100% of patients; the median follow-up time for those alive at the end of the study was 7.4 years.

### Statistical methods

Sample size calculations were performed assuming to achieve a 5-year overall survival of 50%, intermediate between Western and Japanese series. The required number for enrolment was then set to about 200 patients, based on the desired level of power precision in estimating this parameter (95% confidence interval: 42.9–57.1%, power 80%). Confidence intervals are based on exact binomial probabilities. Overall survival was computed by the Kaplan–Meier method using the BMDP statistical package for all eligible subjects and for subpopulations grouped on the basis of selected variables. Both deaths due to the disease and deaths without evidence of recurrence were counted as events in the analysis of survival. The gastric cancer-specific survival curve was also calculated, with deaths due to other causes being censored.

## RESULTS

In total, 297 patients with histologically proven adenocarcinoma of the stomach were registered from the nine institutions over 2½ years (May 1994 – December 1996). Of these, 106 patients were found ineligible for the study mostly because more advanced disease was identified at laparotomy, as outlined in the protocol. In all, 191 patients fulfilled the criteria of eligibility and were entered into the study. Table 1

**Table 1:** Patient characteristics

	**No. of patients (%) (191=100%)**		**No. of patients (%) (191=100%)**
Sex M/F	114 (59.6)/77 (41.4)	IIIB	25 (13.1)
Age <50 years	31 (16.2)	IV	23 (12.1)
Age 50–69 years	103 (53.9)	Pathological stage	
Age 70+ years	57 (29.8)	T1	68 (35.6)
			
Location of tumour		T2	58 (30.3)
Distal third (A,AM)	116 (60.8)	T3	58 (30.3)
Middle third (M,MC,CM)	52 (27.2)	T4	7 (3.8)
Upper third (C,CM)l	13 (6.8)	Nodal status	
More than two-thirds of stomach	6 (3.1)	N0	78 (35.4)
Stump	4 (2.1)	N1	41 (21.5)
			
Japanese stage grouping		N2	56 (29.3)
IA	53 (27.7)	N3 (location no 12)	16 (8.4)
IB	22 (11.5)	Type of gastrectomy	
II	31 (16.2)	Distal	124 (64.9)
IIIA	37 (19.4)	Total	67 (35.1)

briefly summarises the characteristics of the eligible patients (median age: years), the procedures performed, the pathologic stage of the disease and the early outcome.

No patients were lost to follow-up. The median follow-up time of all patients alive at the end of the study was 7.4 years (range 6–8.7 years). All patients were followed up till death or for at least 6 years. Of the 191 resected patients, 96 (50.3%) died. Six out of these 96 patients died with early postoperative complications (3.1%). During the follow-up, 26 patients (13.6%) died without recurrence of gastric cancer. Death with recurrence of gastric cancer occurred in 70 patients (36.7%).

### Decrease of postoperative in-hospital mortality

Postoperative in-hospital mortality may have decreased during the study period. It was 5.26% in 1994 (38 procedures performed), 2.11% in 1995 (95 procedures) and 1.75% in 1996 (57 procedures). Although suggestive of a decreasing trend, due to small numbers percentages are not significantly different from each other (*X*^2^=0.36 (df 2), *P*=0.55; *X*^2^ slope=0.94 (df 1), *P*=0.33).

### Overall survival

For calculating the incidence of deaths due to the disease (*n*=70), the cause of death according to clinical records was used. In those few records where the cause was missing, the cause of death listed in the Piemonte Cancer Registry (from the municipal roster office) was used.

**Figure 1:**
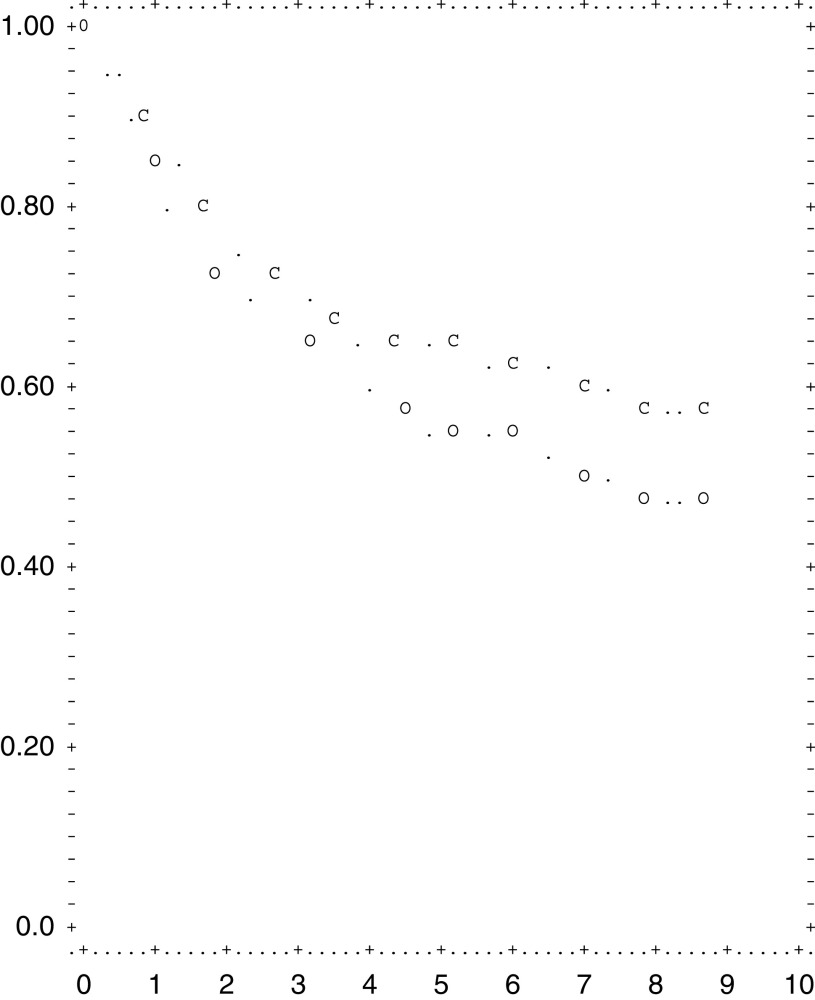
Overall 5-year survival among all eligible. patients (○) and among patients with deaths related to cancer only (C) (95% confidence interval 47.9, 62.1).

The overall 5-year survival rate among all eligible patients was 55.0% (95% confidence interval 47.9, 62.1) (Figure 1

). The gastric cancer specific survival rate was 65% after 5 years and 62.5% after 6 years (Figure 1).

### Survival by TNM stages

**Figure 2:**
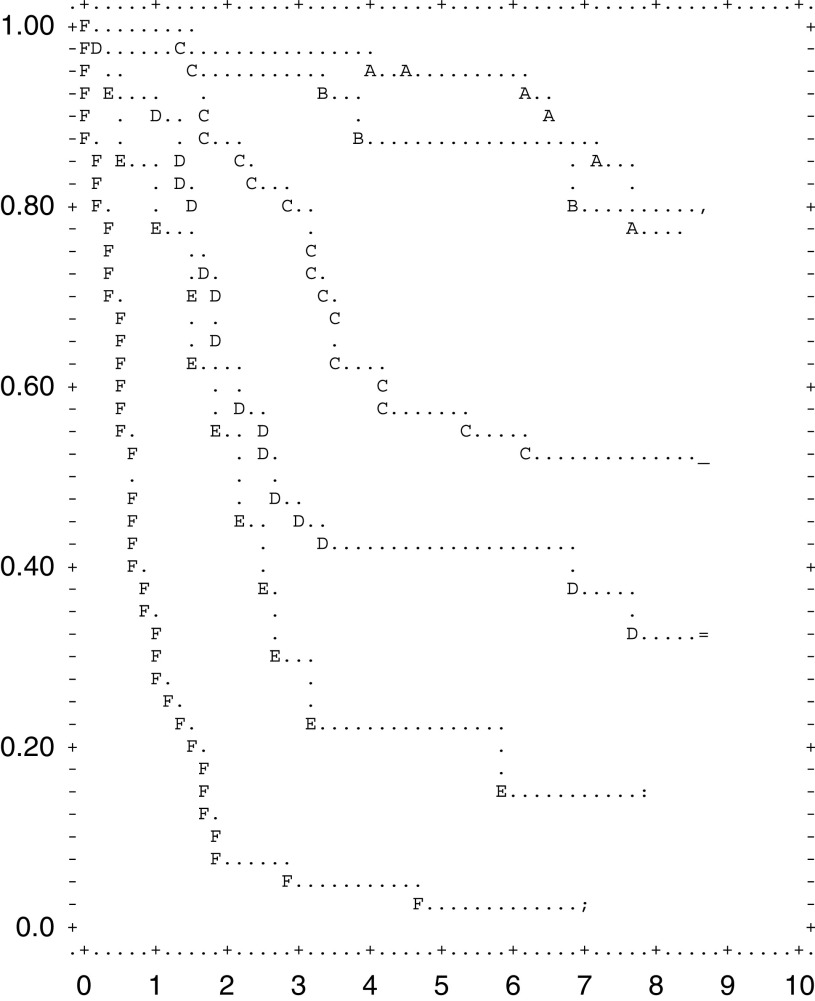
Survival after resection according to 1997 TNM stage (A is IA; B is IB; C is II; D is IIIA; E is IIIB; F is IV).

The 5-year survival rate was significantly dependent upon the stage of the disease (*P*<0.001). It was 95, 87.5, 57.5, 42.5, 22.5 and 2.5% in patients with TNM stage IA, IB, II, IIIA, IIIB and IV, respectively (Figure 2

). To allow comparison of these results with other reports, the results using the previous TNM classification are also shown in Table 2

**Table 2:** Survival among all eligible patients and according to old TNM stage in the most recent series

**Author**	**No of patients**	**Type of gastrectomy (No. of patients)**	**5 years survival (%)**	**IA**	**IB**	**II**	**IIIA**	**IIIB**	**IV**
Wanebo *et al* (1993)	9057	D0–1	26	59	44	29	15	9	3
Siewert *et al*, (1993)	1182^a^	D1 (379)		86	72	26	25	27	28
		D2 (803)		85	68	55	38	17	16
Pacelli *et al* (1993)^b^	238^b^	D2	65	96	73	63	40	33	0
Cuschieri *et al* (1999)	400	D1 (200)	35		69	22		11	Not included
		D2 (200)	33		58	31		11	
Bonenkamp *et al* (1999)^b^	711^b^	D1 (380)	34	81	60	38	11	13	0
		D2 (331)		81	61	42	28	13	28
		D1 (380)	33						
		D2 (331)							
Sasako *et al* (1997)	2541	D2–4	66	92	90	76	59	36	7
IGCSG *et al* (2004)	191	D2	55	92.5	87.5	60	40	20	2.5

a Only Ro resection.

b Only curative resection.

.

### Survival by depth of invasion

**Figure 3:**
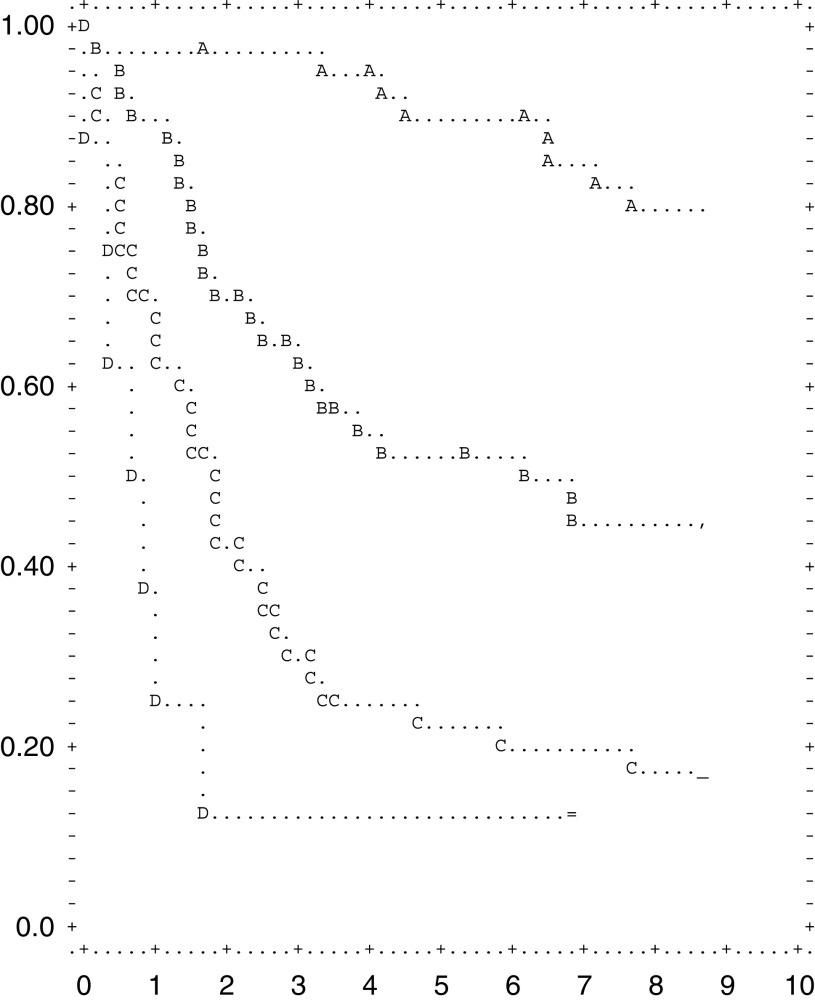
Survival by depth of invasion (pT) (A is T1; B is T2; C is T3; D is T4).

Survival of patients was significantly influenced by depth of invasion (*P*<0.001). The 5-year survival rate was 90, 52.5, 25 and 12.5 for patients with T1, T2, T3 and T4, respectively (Figure 3

).

### Survival by nodal involvement

We analysed patient survival according to the two nodal staging systems: the 1997 TNM and the 1998 JGCA classification.

**Figure 4:**
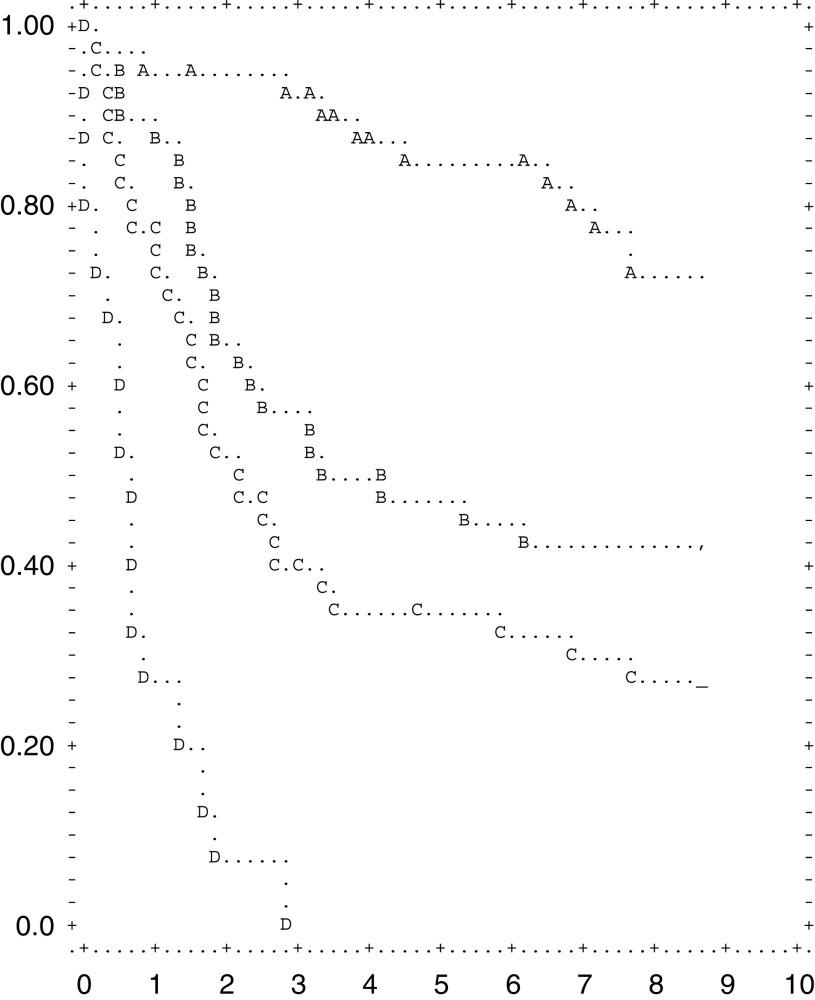
Survival by JGCA nodal involvement (A is N0;B is N1; C is N2; D is N3).

The. 5-year survival rates of pN0, pN1, pN2 and pN3 by 1997 TNM were 85, 52.5, 32.5 and 2.5%, respectively. Those by the JGCA classification were 47.5%, 35 % and 0% for pN1, pN2 and pN3, respectively (Figure 4

).

### Survival by type of gastrectomy

Patients who underwent distal gastrectomy showed a higher 5-year survival rate (70%) as compared with those who received total resection (40%) (*P*<0.001).

## DISCUSSION

The role of the extended lymph node dissection in improving long-term survival after gastrectomy for gastric cancer is still not proven by RCTs. Moreover, the Dutch and British trials have shown increased morbidity and mortality figures after D2 gastrectomy (Bonenkamp *et al*, 1995; Cuschieri *et al*, 1996). Potential reasons for this unfavourable outcome include the lack of surgical skilfulness/training and poor quality control, and the routine removal of the spleen and tail of the pancreas in total gastrectomy (Cuschieri *et al*, 1996).

In our previous paper, we showed that it is possible to achieve low morbidity and mortality after extended lymph node dissection, if the operation is performed in specialised centres with a strict quality control system, and without removing the pancreas during total gastrectomy unless it is suspected to be involved by the tumour (Degiuli *et al*, 1998).

The present study has also shown good survival data. The overall 5-year survival rate was 55%. Moreover, the disease-specific 5-year survival was 65%. Our results are almost equivalent to those reported by Sasako after 2541 extended gastrectomies performed at the National Cancer Center Hospital, Tokyo, during the period ‘1982–1991’ (66%) (Sasako *et al*, 1997, pp 223–248). Not only the overall survival rate but also the stage-specific survival rates after D2 dissection were much better in this study than those of the D2 arm of the Dutch and MRC trials (Table 2).

The discrepancy between our data and data from other Western series could be explained by differences in the patient population or by differences in surgical technique.

Regarding the patient populations, the eligibility criteria from the two large prospective randomised series are totally comparable to those adopted in our trial.

With respect to the clinical and pathological stages, no major differences appear in the reported series apart from a clear prevalence of early gastric cancer in the Japanese series. The prevalence of early tumours (stage I disease) is close to 50% in the Japanese series, while it is 35.6% in our population, 36% in the MRC series, 26% in the Dutch trial and 19.6% in an American patient care study (16). Siewert gives the figures for IA and IB stages, which are, respectively, 13.8 and 13.4% (3). In the present series, the number of patients with TNM stage less than III is substantial (106 patients, 55.4%) and might be partly responsible for our good survival data.

To avoid the confounding effect of stage migration, we should compare the results of series reporting D2 dissection with each other. Our results are similar to those previously reported by Pacelli *et al*. (1993) in their retrospective trial and by Siewert *et al* (1993) in their prospective nonrandomised trial.

The main criticism that has been directed towards the recent prospective randomised European trials has been the lack of experience of the surgeons participating in the study. The contrast in postoperative mortality between the Dutch or British trials and our own study clearly demonstrated the danger of carrying out this procedure, let alone an RCT, without sufficient pretrial training. Clearly a one-arm study, equivalent to the phase II study in medical treatment, is an appropriate preliminary to a phase III trial of complex and potentially hazardous surgery. MS, who was supervisor of both the Dutch and the Italian study, believes that the Dutch study was flawed by early randomisation of patients, and the inclusion of many small-volume hospitals. It is suggested that a new surgical technique requiring not only surgical skills but also good experience in postoperative care should only be tested in an RCT after completion of sufficient training to carry it out safely. In fact, the reported perioperative mortalities in these two major RCTs on D2 dissection were over 10%. Pancreaticoduodenectomy for pancreatic cancer or radical oesophagectomy for oesophageal cancer are more surgically aggressive procedures than D2 gastrectomy and are recommended to be performed exclusively in specialised centres. They do not carry a risk of hospital mortality of over 10% in such centres (Altorki and Skinner, 1997; Gordon *et al*, 1998; Bottger and Junginger, 1999; Lerut *et al*, 1999; Tsiotos *et al*, 1999; Gouma *et al*, 2000; Karl *et al*, 2000). Postoperative mortality of over 10% is no longer acceptable in any kind of cancer surgery.

Our own experience correlates well with the data given by Parikh *et al* (1996) about the duration of the learning curve for D2 dissection, which should be more than 15 procedures. Each participating centre treated 15 to more than 25 patients (seven procedures per year on an average) (Table 3

**Table 3:** Relative experience of participating centres in Italian, British and Dutch trials.

	**IGCSG**	**DGCG^a^**	**MRC^b^**
No. of centres	9	80	322
No. of surgeons	9 pairs	11/85^c^	32
Duration of enrolment (years)	2.5	4	7
No of patients	191	331	200
Average no. of procedures/hospital/year	7	1.5	1

a Dutch Gastric Cancer Group trial.

b Medical Research Council, British Trial.

c Supervising/local surgeons.

), and in every centre each patient was always treated by the same two surgeons. Therefore, each centre and each surgeon should have reached an optimal experience level, acquiring sufficient technical skills regarding intra- and postoperative care during this trial. Our results support the argument for training the surgeons prior to the initiation of a clinical trial although, at a practical level, a study target of 700–1000 patients would be very difficult to conduct, and it might take more than 10 years to recruit all the patients.

We observed an overall postoperative in-hospital mortality of 3.1%: this rate has been decreasing from 5.2% in 1994, to 2.11% in 1995 and finally to 1.7% in 1996. While not statistically significant, this trend supports the concept of a learning curve.

As already indicated, subset analysis of the Dutch and MRC trials documented that the higher morbidity in the D2 arm is mostly due to pancreas and spleen removal (Cuschieri *et al*, 1996). Hence, pancreas preservation was adopted as standard procedure in D2 dissection in the present trial. Therefore, the pancreas was removed only when it was suspected to be involved by the tumour (T4). Furthermore, during total gastrectomy, splenectomy was not carried out in patients with clinical T1 tumour (Table 4

**Table 4:** Spleen and pancreas removal during total gastrectomies in Italian, British and Dutch trials

	**IGCSG no. (%)**	**DGCG^a^ no. (%)**	**MRC^b^ no. (%)**
Total gastrectomies	67 (100)	126 (100)	108 (100)
Splenectomies	49 (73.1)	124 (98.4)	131 (121.2)^c^
Pancreatectomies	10 (14.9)	98 (77.7)	113 (104.6)^d^

a Medical Research Council, British trial.

b Dutch Gastric Cancer Group trial.

c A total of 25 splenectomies performed during a distal gastrectomy.

d Five pancreatectomies performed during a distal gastrectomy.

).

After confirming the low mortality and acceptable morbidity of pancreas-preserving D2 dissection, we started a phase III trail, comparing D1 *vs* D2 in 1998. The survival results shown in this paper suggest the benefits of D2 dissection, although a statistically significant survival advantage needs to be confirmed through this new randomised phase III trial. The aim of this phase III trial is to document an increase of survival in the D2 arm with acceptable increase of morbidity and without increase of mortality.
